# *Helicobacter pylori* infection, hyperuricemia, and serum uric acid levels: The role of metabolic, renal, and inflammatory factors in a large health examination population

**DOI:** 10.1371/journal.pone.0358039

**Published:** 2026-09-10

**Authors:** Zhiyue Xu, Xiuyu Wang, Jie Huang, Chunxing Liu, Yong Fang

**Affiliations:** 1 Department of Nursing, Shanghai Health and Medical Center, Wuxi, Jiangsu Province, China; 2 Department of Diagnostic Imaging, Shanghai Health and Medical Center, Wuxi, Jiangsu Province, China; 3 Department of Clinical Laboratory, Shanghai Health and Medical Center, Wuxi, Jiangsu Province, China; Helwan University, EGYPT

## Abstract

**Background:**

*Helicobacter pylori* (*H. pylori*) infection has been linked to several extragastric metabolic disorders. However, its cross-sectional association with hyperuricemia remains uncertain, partly because previous studies have often been limited by small sample sizes and incomplete adjustment for metabolic and renal factors.

**Methods:**

This large-scale cross-sectional study used data from a health examination database. The main regression analyses included 67,826 participants with valid *H. pylori* status, serum uric acid measurements, hyperuricemia classification, age, sex, and BMI. Modified Poisson regression with a log link and HC0 robust sandwich variance was used to estimate prevalence ratios for hyperuricemia, while linear regression with HC3 heteroscedasticity-robust standard errors was used for continuous serum uric acid. Sex-stratified and robustness analyses were also performed.

**Results:**

In the unadjusted model, *H. pylori* infection was associated with higher prevalence of hyperuricemia (PR = 1.27, 95% CI: 1.22–1.32, *p* < 0.001). This association was progressively attenuated after sequential adjustment and was small in the extended clinical model (PR = 1.02, 95% CI: 0.98–1.06, *p* = 0.269). For continuous serum uric acid, *H. pylori* positivity remained associated with a small increase after full adjustment (*β* = 2.14 μmol/L, 95% CI: 0.80–3.49, *p* = 0.002). Functional-form sensitivity analyses yielded similarly small effect estimates for hyperuricemia, although statistical significance varied across model specifications. Sex-stratified and robustness analyses were generally consistent with these findings.

**Conclusion:**

In this large health examination population, any remaining association between H. pylori positivity and hyperuricemia after comprehensive adjustment was small and sensitive to model specification. *H. pylori* positivity was also associated with a statistically detectable but clinically modest difference in continuous serum uric acid levels. Given the cross-sectional design, these findings should not be interpreted causally.

## 1. Introduction

*Helicobacter pylori* (*H. pylori*) infection is one of the most common chronic bacterial infections worldwide and is well established as a major cause of chronic gastritis, peptic ulcer disease, and gastric cancer [1]. In addition to its gastric manifestations, *H. pylori* infection has been investigated in relation to several extragastric conditions, including metabolic abnormalities, low-grade systemic inflammation, and cardiometabolic risk [2]. Observational studies and meta-analyses have suggested that *H. pylori* infection may be associated with metabolic syndrome and insulin resistance, although the evidence remains inconsistent across populations [3]. The substantial heterogeneity reported in previous studies suggests that these associations may depend on population characteristics, clinical background, and the extent to which metabolic and inflammatory confounders are controlled [3].

Hyperuricemia is a common metabolic abnormality and represents the key biochemical basis of gout. It is also closely linked to obesity, insulin resistance, elevated blood pressure, impaired renal function, and cardiometabolic disease risk [4,5]. Serum uric acid homeostasis is determined by the balance between urate production and excretion, with renal excretion playing a central role in maintaining circulating uric acid levels [6]. Experimental and clinical studies have shown that insulin resistance and hyperinsulinemia can reduce renal uric acid excretion and promote urate reabsorption, thereby contributing to hyperuricemia [7,8]. Renal tubular urate transporters, including URAT1 and GLUT9, are also critical regulators of urate reabsorption and serum uric acid homeostasis [6,9]. Therefore, if *H. pylori* infection is intertwined with insulin resistance, chronic inflammation, or renal functional changes, it may plausibly be associated with serum uric acid levels and hyperuricemia.

However, the adjusted cross-sectional association between *H. pylori* infection and abnormalities in uric acid metabolism remains uncertain. Some observational studies have reported that *H. pylori* infection is associated with higher serum uric acid levels or a greater burden of uric acid–related outcomes, whereas others suggest that these associations may be substantially attenuated after adjustment for metabolic and renal factors [10,11]. Chen et al. reported a positive association between *H. pylori* infection and gout among Chinese adults with hyperuricemia, whereas Wang et al. observed higher serum uric acid levels in crude comparisons but no significant overall association after multivariable adjustment, with evidence of effect modification by renal function [10,11]. Our study extends this literature by examining both hyperuricemia and continuous serum uric acid in a substantially larger health-examination population with detailed renal and metabolic adjustment and functional-form sensitivity analyses.

In this context, the present study used data from a large health examination population to systematically evaluate the cross-sectional associations of *H. pylori* infection with hyperuricemia and continuous serum uric acid levels. Using sequentially adjusted models, we examined the extent to which demographic factors, BMI, blood pressure, glucose and lipid metabolism, renal function, liver enzymes, and systemic inflammatory markers accounted for these associations. We also performed sex-stratified analyses and multiple robustness analyses to assess the consistency and clinical relevance of the observed cross-sectional associations between *H. pylori* infection and abnormalities in uric acid metabolism.

## 2. Methods

### 2.1. Study design and Data Source

This retrospective cross-sectional study was approved by the Ethics Committee of Shanghai Health and Medical Center (approval number: (2026) No. 5; approval date: March 2, 2026) and used deidentified routine health-examination records maintained by Shanghai Health and Medical Center, Wuxi, Jiangsu Province, China. The study was conducted in accordance with the Declaration of Helsinki. The present secondary analysis was confirmed to fall within the scope of the approved deidentified health examination dataset. The study period extended from October 1, 2024, to October 31, 2025, and the deidentified dataset was accessed for research purposes on May 21, 2026. The requirement for informed consent was waived because this was a retrospective analysis of deidentified data. Participants were identified using an anonymized personal identifier to avoid duplicate inclusion of repeated examinations; for participants with more than one health examination record during the study period, only the most recent valid examination record was retained to construct an individual-level analytical dataset. The authors did not have access to direct personal identifiers during or after data collection.

### 2.2. Study Population

**Fig 1:**
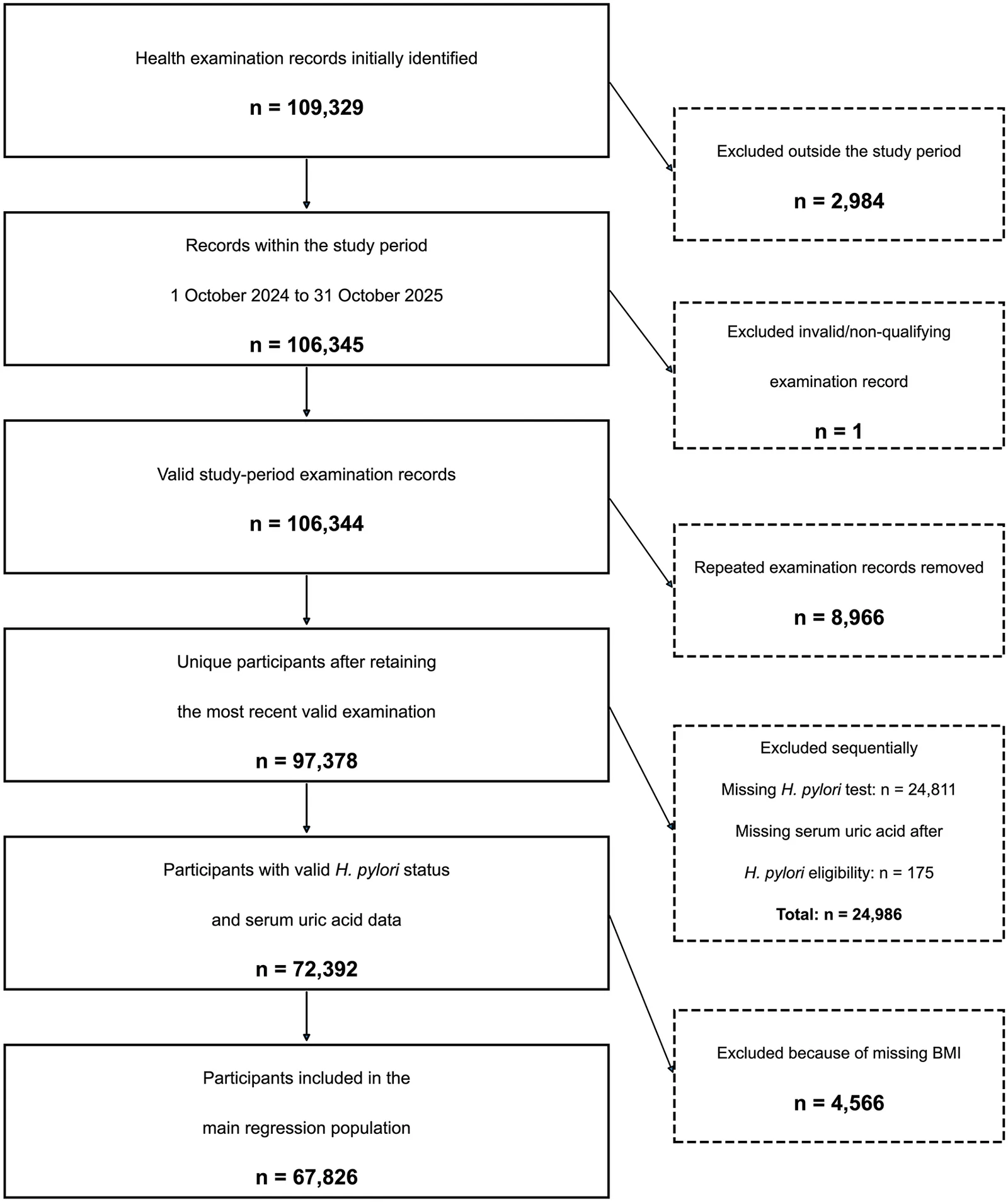
Flow diagram of participant selection.

The original database contained 109,329 health examination records. After restricting the records to the study period, 106,345 records remained. One non-qualifying examination record with essentially unavailable examination measurements was excluded before participant-level deduplication, leaving 106,344 valid study-period records. For participants with repeated examinations, only the most recent valid examination record was retained, yielding 97,378 unique participants. We then excluded 24,811 participants with missing *H. pylori* test results and, among the remaining participants, 175 with missing serum uric acid measurements, leaving 72,392 participants for the baseline analyses. After excluding 4,566 participants with missing BMI, 67,826 participants remained for the main regression analyses; no participants were excluded because of missing age or sex at this stage (Fig 1).

### 2.3. Definition of *H. pylori* Infection

*H. pylori* infection status was determined using the fasting ^13^C-urea breath test (^13^C-UBT), which was routinely performed as part of health-examination services at the study center rather than specifically administered for this study. Participants undergoing testing fasted for at least 8 h. A baseline (0-min) breath sample was collected by exhaling through a straw into a sample bag for approximately 4–5 s. Participants then ingested 75 mg of ^13^C-urea granules (Beijing Boran Pharmaceutical Co., Ltd., Beijing, China) with 80 mL of cool water and remained seated for 30 min, after which a second breath sample was collected using the same procedure. Breath samples were analyzed using a HY-IREXplus breath analyzer (Jiangxi Xinyu Lian’er Medical Technology Co., Ltd., Jiangxi, China). Routine instrument self-checks were performed in accordance with the analyzer operating procedures. Results were expressed as delta over baseline (DOB), calculated as the difference between the 30-min and baseline measurements. In accordance with the manufacturer’s recommended decision threshold, a DOB ≥ 4.0 was classified as *H. pylori* positive and a DOB < 4.0 as negative. Participants without an available *H. pylori* test result were excluded from the corresponding analyses.

### 2.4. Definition of Serum Uric Acid and Hyperuricemia

Serum uric acid was analyzed as a continuous outcome. Hyperuricemia was analyzed as the primary binary outcome and was defined using conventional sex-specific epidemiological thresholds of serum uric acid >420 μmol/L in men and >360 μmol/L in women [12].

### 2.5. Laboratory Measurements

After an overnight fast of 8–12 h, venous blood samples were collected during the health examinations. Serum biochemical measurements, including uric acid, fasting glucose, lipid parameters, liver enzymes, creatinine, and hs-CRP, were performed on a Roche cobas 8000 analytical platform (c702 chemistry module; Roche Diagnostics) using manufacturer-matched Roche reagents. Serum uric acid was measured using a uricase/peroxidase enzymatic colorimetric method, fasting glucose using a hexokinase UV method, triglycerides using an enzymatic colorimetric LPL/GK/GPO/POD method, and HDL-C using a homogeneous enzymatic colorimetric method. ALT and AST were measured using IFCC-traceable kinetic UV methods based on LDH- and MDH-coupled NADH oxidation, respectively; serum creatinine was measured using an enzymatic creatinine method, and GGT using an enzymatic colorimetric rate method. White blood cell count and hemoglobin were measured using a BC-6800Plus automated hematology analyzer on a CAL 8000Plus line (Mindray Bio-Medical Electronics, Shenzhen, China). WBC counting and classification were based on laser scattering with fluorescent staining flow cytometry, whereas hemoglobin was measured using a colorimetric absorbance method. Routine calibration and internal quality-control procedures were performed in accordance with laboratory standard operating procedures and manufacturer instructions.

### 2.6. Covariates

Covariates were selected by clinical domain, prior epidemiological evidence, measurement availability, parsimony, and avoidance of redundant correlated measures; selection was not based on *p* values or stepwise procedures. The basic adjustment model included age, sex, and BMI. BMI was used as the primary general adiposity measure, so waist circumference was not simultaneously entered. The metabolic adjustment model further included systolic blood pressure, fasting blood glucose, triglycerides, and HDL-C. SBP was used as the principal blood-pressure indicator, so DBP was not simultaneously entered. FBG was used as the primary routinely available glycemic measure because HbA1c had greater missingness and was not required for the primary model. The extended clinical model additionally included eGFR, ALT, GGT, and WBC. ALT and GGT were included as complementary hepatic/metabolic indicators, whereas AST was not simultaneously included to limit redundant liver-enzyme adjustment. The sequential adjustment framework was based on clinical domains and available epidemiological evidence; additional assumption and robustness analyses were conducted to examine model assumptions.

HDL-C, liver enzymes, renal function indices, and WBC were treated as available clinical indicators reflecting metabolic, hepatic, renal, and inflammatory status. However, these variables could not fully replace direct information on diet, alcohol intake, smoking, physical activity, or medication use. Medication use, including urate-lowering agents, diuretics, antibiotics, proton pump inhibitors, and bismuth-containing medications, was not used as an exclusion criterion because individual-level medication information was not available in the retrospective database.

eGFR was calculated using the 2021 race-free Chronic Kidney Disease Epidemiology Collaboration (CKD-EPI) creatinine equation, which incorporates serum creatinine, age, and sex. Serum creatinine values were converted from μmol/L to mg/dL before calculation [13]. Because hs-CRP had substantial missingness, it was not included in the primary extended clinical model and was evaluated only in a sensitivity analysis among participants with available hs-CRP data.

### 2.7. Statistical Analysis

The distribution of continuous variables was assessed using histograms, Q-Q plots, and the Kolmogorov–Smirnov test. Approximately normally distributed variables were summarized using means ± standard deviations and compared using Welch’s t test. Markedly skewed variables were summarized using medians and interquartile ranges (IQRs) and compared using the Mann–Whitney U test. Categorical variables were summarized as *n* (%) and compared using the chi-square test. Absolute standardized mean differences (SMDs) were calculated for baseline comparisons; continuous SMDs used the difference in group means divided by the pooled standard deviation, and binary standardized differences were used for dichotomous variables.

Hyperuricemia was analyzed as the primary binary outcome using modified Poisson regression with a log link and HC0 robust sandwich variance; results were expressed as prevalence ratios (PRs) and 95% confidence intervals (CIs). Logistic regression was retained as a supplementary sensitivity analysis, with results expressed as odds ratios (ORs). Continuous serum uric acid was analyzed using ordinary least-squares linear regression, with HC3 heteroscedasticity-robust standard errors used for CIs and *p* values. *β* represents the adjusted mean difference in serum uric acid associated with *H. pylori* positivity compared with *H. pylori* negativity.

Sequential Models 1–4 were used to evaluate progressive attenuation and were not an automated stepwise variable-selection procedure. Model 1 was unadjusted. Model 2 was adjusted for age, sex, and BMI. Model 3 was further adjusted for systolic blood pressure, fasting blood glucose, triglycerides, and HDL-C. Model 4 was the extended clinical model and was further adjusted for eGFR, ALT, GGT, and WBC. Additional sensitivity analyses were conducted to examine model assumptions, including a 4-knot all-continuous restricted cubic spline analysis using the 5th, 35th, 65th, and 95th percentiles; a 3-knot placement sensitivity analysis using the 10th, 50th, and 90th percentiles; and joint log transformation of FBG, TG, ALT, and GGT. These additional analyses were not selected according to statistical significance. The alpha level for statistical significance was 0.05.

Sex-stratified analyses were performed to evaluate the associations of *H. pylori* infection with hyperuricemia and continuous serum uric acid levels separately in men and women. Sex-stratified models were adjusted for age, BMI, systolic blood pressure, fasting blood glucose, triglycerides, HDL-C, eGFR, ALT, GGT, and WBC. To formally assess sex interaction, full-population models including *H. pylori* infection, sex, an *H. pylori* infection × sex interaction term, and the same covariates were fitted.

Robustness analyses included: (1) replacing eGFR with serum creatinine; (2) repeating the analyses after excluding participants with eGFR < 60 mL/min/1.73 m²; (3) repeating the analyses after excluding participants with serum uric acid values below the 1st percentile or above the 99th percentile; and (4) additionally adjusting for hs-CRP in the hs-CRP complete-case subset. WBC was retained in the primary model as a widely available inflammation-related marker, whereas hs-CRP was evaluated in a sensitivity analysis. Outliers in serum uric acid were not removed from the primary analysis; their influence was evaluated by excluding values outside the 1st to 99th percentiles in sensitivity analysis.

To assess multicollinearity among covariates in the extended clinical model, variance inflation factors (VIFs) were calculated. A VIF value <5 was considered to indicate no substantial multicollinearity. Interaction by sex was assessed using a multiplicative *H. pylori* infection × sex term in full-population models. All statistical tests were two-sided, and *p* < 0.05 was considered statistically significant. Exact *p* values were reported when *p* ≥ 0.001, whereas smaller values were reported as *p* < 0.001. No correction for multiple comparisons was applied because the analyses addressed the two study outcomes and model-assumption sensitivity rather than screening a large set of unrelated hypotheses. Analyses were performed using Python 3.13.9 with pandas 2.3.3, NumPy 2.3.5, SciPy 1.16.3, statsmodels 0.14.5, and patsy 1.0.1. Modified Poisson models were fitted using statsmodels generalized linear models with a Poisson family, log link, and HC0 robust covariance. Continuous-outcome models were fitted using ordinary least-squares regression with HC3 heteroscedasticity-robust covariance. Restricted cubic splines were constructed using patsy. Complete regression results and supporting sensitivity analyses are provided in the Supporting Information.

### 2.8. Handling of Missing Data

Missing data were evaluated separately in the deduplicated population, the baseline eligible population, and the model-specific analysis populations. Among the 72,392 baseline eligible participants, 4,566 (6.31%) had missing BMI; no participants had missing age or sex at this stage. These participants were excluded, leaving 67,826 participants for the main regression population. Each regression model used complete cases for the variables included in that model. Additional missingness in Model 3 covariates reduced the sample size to 67,540, and additional missingness in Model 4 covariates reduced it to 67,522. Variable-level missingness is reported in S2 Table.

hs-CRP measurements were available for 25,928 of 67,826 participants (38.23%) in the main regression population. Because of substantial missingness, hs-CRP was not included in primary models. The hs-CRP sensitivity analysis compared the fully adjusted Model 4 with and without hs-CRP in the same fixed complete-case subset, which included 25,828 participants.

## 3. Results

### 3.1. Baseline characteristics of the study population

Fig 1 summarizes participant selection. Of 109,329 health examination records initially identified, 106,345 were within the study period. One non-qualifying examination record with essentially unavailable examination measurements was excluded before participant-level deduplication, leaving 106,344 valid study-period records. After retaining only the most recent valid examination for participants with repeated records, 97,378 unique participants remained. Sequentially excluding 24,811 participants with missing *H. pylori* test results and 175 participants with missing serum uric acid measurements yielded a baseline population of 72,392. Exclusion of 4,566 participants with missing BMI yielded the main regression population of 67,826.

Among the baseline population presented in Table 1, 61,739 participants were *H. pylori* negative and 10,653 were *H. pylori* positive. Compared with *H. pylori*-negative participants, *H. pylori*-positive participants had a higher proportion of men (68.3% vs 60.2%, *p* < 0.001) and higher BMI, waist circumference, systolic blood pressure, and diastolic blood pressure (all *p* < 0.001).

**Table 1:** Baseline characteristics by *H. pylori* infection status.

Variable	*H. pylori* negative (*n* = 61,739)	*H. pylori* positive (*n* = 10,653)	SMD	*p* value
Age, years	44.79 ± 11.82	45.73 ± 11.70	0.080	<0.001
Male sex, *n* (%)	37,189 (60.2%)	7,279 (68.3%)	0.169	<0.001
BMI, kg/m²	24.56 ± 3.52	25.34 ± 3.70	0.220	<0.001
Waist circumference, cm	82.31 ± 11.27	84.90 ± 10.72	0.232	<0.001
SBP, mmHg	119.88 ± 15.38	122.60 ± 16.22	0.175	<0.001
DBP, mmHg	73.27 ± 10.60	75.18 ± 11.24	0.179	<0.001
Uric acid, μmol/L	334.08 ± 82.45	349.94 ± 84.99	0.191	<0.001
Hyperuricemia	10,776 (17.5%)	2,352 (22.1%)	0.116	<0.001
FBG, mmol/L	5.37 ± 0.94	5.56 ± 1.29	0.191	<0.001
HbA1c, %	5.42 ± 0.65	5.55 ± 0.87	0.189	<0.001
TG, mmol/L	1.16 [0.82, 1.73]	1.32 [0.90, 2.01]	0.161	<0.001
TC, mmol/L	4.87 ± 0.92	4.97 ± 0.97	0.107	<0.001
HDL-C, mmol/L	1.36 ± 0.37	1.29 ± 0.36	0.176	<0.001
LDL-C, mmol/L	3.09 ± 0.82	3.17 ± 0.86	0.105	<0.001
ALT, U/L	18.00 [12.00, 27.00]	19.00 [13.00, 29.00]	0.107	<0.001
AST, U/L	19.64 ± 11.67	20.41 ± 11.31	0.066	<0.001
GGT, U/L	21.00 [14.00, 34.00]	24.00 [15.00, 40.00]	0.174	<0.001
Creatinine, μmol/L	75.78 ± 17.25	78.30 ± 26.78	0.132	<0.001
eGFR, mL/min/1.73 m²	101.29 ± 13.84	99.79 ± 14.31	0.108	<0.001
eGFR < 60	336 (0.5%)	87 (0.8%)	0.033	<0.001
BUN, mmol/L	5.47 ± 1.28	5.49 ± 1.36	0.018	0.107
WBC, 10⁹/L	6.14 ± 1.53	6.56 ± 1.66	0.270	<0.001
Hemoglobin, g/L	144.58 ± 15.41	146.90 ± 15.91	0.150	<0.001
hs-CRP, mg/L	0.74 [0.36, 1.62]	0.85 [0.40, 1.95]	0.086	<0.001
Hcy, μmol/L	11.10 [9.50, 12.90]	11.70 [10.00, 13.80]	0.180	<0.001

Note: Values are presented as mean ± SD, median [Q1, Q3], or *n* (%), as appropriate. Absolute standardized mean differences are shown. For continuous variables, SMDs were calculated as the difference in group means divided by the pooled standard deviation; binary standardized differences were used for dichotomous variables. Variable-specific denominators were used where measurements were missing. *p* values were calculated using Welch’s t test, the Mann–Whitney U test, or the chi-square test, as appropriate. hs-CRP was available only in a subset of participants. BMI, body mass index; SBP, systolic blood pressure; DBP, diastolic blood pressure; eGFR, estimated glomerular filtration rate; FBG, fasting blood glucose; HbA1c, glycated hemoglobin; TG, triglycerides; TC, total cholesterol; HDL-C, high-density lipoprotein cholesterol; LDL-C, low-density lipoprotein cholesterol; ALT, alanine aminotransferase; AST, aspartate aminotransferase; GGT, gamma-glutamyl transferase; BUN, blood urea nitrogen; WBC, white blood cell count; hs-CRP, high-sensitivity C-reactive protein; Hcy, homocysteine.

*H. pylori*-positive participants also had higher serum uric acid levels than *H. pylori*-negative participants (349.94 ± 84.99 vs 334.08 ± 82.45 μmol/L, *p* < 0.001), as well as a higher prevalence of hyperuricemia (22.1% vs 17.5%, *p* < 0.001). In addition, *H. pylori*-positive participants showed higher fasting blood glucose, HbA1c, triglycerides, ALT, GGT, creatinine, WBC, hs-CRP, and homocysteine levels, whereas HDL-C and eGFR levels were lower (all *p* < 0.001).

### 3.2. Association Between H. pylori Infection and Hyperuricemia

In the main analysis population, the prevalence of hyperuricemia was higher among *H. pylori*-positive participants than among *H. pylori*-negative participants (22.23% vs 17.49%, *p* < 0.001). In the unadjusted modified Poisson model, *H. pylori* infection was associated with higher prevalence of hyperuricemia (PR = 1.27, 95% CI: 1.22–1.32, *p* < 0.001).

After adjustment for age, sex, and BMI, the association was attenuated but remained statistically significant (PR = 1.09, 95% CI: 1.05–1.13, *p* < 0.001). Further adjustment for systolic blood pressure, fasting blood glucose, triglycerides, and HDL-C led to additional attenuation (PR = 1.06, 95% CI: 1.02–1.11, *p* = 0.002). In the extended clinical model, which additionally included eGFR, ALT, GGT, and WBC, the association was small and the confidence interval included the null (PR = 1.02, 95% CI: 0.98–1.06, *p* = 0.269). Progressive attenuation therefore remained evident across the sequential models. The corresponding model estimates are shown in Table 2.

**Table 2:** Association between *H. pylori* infection and hyperuricemia estimated using modified Poisson regression with robust variance.

Model	Adjustment	N	PR	95% CI	*p* value
Model 1	Unadjusted	67,826	1.27	1.22–1.32	<0.001
Model 2	Age, sex, BMI	67,826	1.09	1.05–1.13	<0.001
Model 3	Model 2 + SBP, FBG, TG, HDL-C	67,540	1.06	1.02–1.11	0.002
Model 4	Model 3 + eGFR, ALT, GGT, WBC	67,522	1.02	0.98–1.06	0.269

Note: Model 1 was unadjusted. Model 2 was adjusted for age, sex, and BMI. Model 3 was further adjusted for systolic blood pressure, fasting blood glucose, triglycerides, and HDL-C. Model 4 was the extended clinical model and was further adjusted for eGFR, ALT, GGT, and WBC. PRs were estimated using modified Poisson regression with a log link and HC0 robust sandwich variance. PR, prevalence ratio; CI, confidence interval; BMI, body mass index; SBP, systolic blood pressure; FBG, fasting blood glucose; TG, triglycerides; HDL-C, high-density lipoprotein cholesterol; eGFR, estimated glomerular filtration rate; ALT, alanine aminotransferase; GGT, gamma-glutamyl transferase; WBC, white blood cell count.

These findings indicate that the crude cross-sectional association was progressively attenuated after adjustment for measured metabolic, renal, hepatic, and inflammatory factors; the fully adjusted estimate should be interpreted as small and model-dependent rather than as evidence of a causal relationship.

### 3.3. Association Between *H. pylori* Infection and Continuous Serum Uric Acid Levels

In the analysis of continuous serum uric acid, *H. pylori*-positive participants had higher mean serum uric acid levels than *H. pylori*-negative participants (350.76 ± 84.86 vs 334.62 ± 82.46 μmol/L, *p* < 0.001). In the unadjusted linear regression model, *H. pylori* positivity was associated with a 16.14 μmol/L higher serum uric acid level (95% CI: 14.34–17.94, *p* < 0.001).

After adjustment for age, sex, and BMI, the difference was substantially reduced (*β* = 4.37 μmol/L, 95% CI: 2.95–5.80, *p* < 0.001). Further adjustment for blood pressure, glucose metabolism, and lipid metabolism yielded a *β* coefficient of 3.70 μmol/L (95% CI: 2.30–5.09, *p* < 0.001). In the extended clinical model, which additionally included eGFR, ALT, GGT, and WBC, *H. pylori* positivity remained associated with a modest increase in serum uric acid level (*β* = 2.14 μmol/L, 95% CI: 0.80–3.49, *p* = 0.002).

Although the fully adjusted association was statistically detectable, the effect magnitude was small and of limited clinical relevance.

Detailed continuous-outcome estimates are presented in Table 3.

**Table 3:** Association between *H. pylori* infection and serum uric acid levels estimated using OLS with HC3 robust inference.

Model	Adjustment	N	*β* (μmol/L)	95% CI	*p* value
Model 1	Unadjusted	67,826	16.14	14.34–17.94	<0.001
Model 2	Age, sex, BMI	67,826	4.37	2.95–5.80	<0.001
Model 3	Model 2 + SBP, FBG, TG, HDL-C	67,540	3.70	2.30–5.09	<0.001
Model 4	Model 3 + eGFR, ALT, GGT, WBC	67,522	2.14	0.80–3.49	0.002

Note: Model 1 was unadjusted. Model 2 was adjusted for age, sex, and BMI. Model 3 was further adjusted for systolic blood pressure, fasting blood glucose, triglycerides, and HDL-C. Model 4 was the extended clinical model and was further adjusted for eGFR, ALT, GGT, and WBC. *β* coefficients represent the adjusted mean difference in serum uric acid associated with *H. pylori* positivity. 95% CIs and *p* values were calculated using HC3 heteroscedasticity-robust standard errors. CI, confidence interval; BMI, body mass index; SBP, systolic blood pressure; FBG, fasting blood glucose; TG, triglycerides; HDL-C, high-density lipoprotein cholesterol; eGFR, estimated glomerular filtration rate; ALT, alanine aminotransferase; GGT, gamma-glutamyl transferase; WBC, white blood cell count.

### 3.4. Sex-Stratified and Interaction Analyses

In the sex-stratified analyses, the prevalence of hyperuricemia was higher among *H. pylori*-positive than *H. pylori*-negative participants in both men (28.17% vs 24.25%) and women (8.92% vs 6.96%). Among men, the HUA PR was 1.10 (95% CI: 1.06–1.15, *p* < 0.001) in the basic adjusted model and 1.04 (95% CI: 0.99–1.08, *p* = 0.087) in the extended clinical model. Among women, the corresponding PRs were 1.02 (95% CI: 0.90–1.15, *p* = 0.794) and 0.96 (95% CI: 0.85–1.08, *p* = 0.509). The *H. pylori* infection × sex interaction was not statistically significant (*p* = 0.110).

For continuous serum uric acid levels, the extended clinical *β* was 2.60 μmol/L among men (95% CI: 0.86–4.35, *p* = 0.004) and 1.33 μmol/L among women (95% CI: −0.59–3.26, *p* = 0.174). The *H. pylori* infection × sex interaction was not statistically significant (*p* = 0.765).

Overall, although the association appeared slightly stronger in men, formal interaction tests did not support sex-specific heterogeneity in the associations of *H. pylori* infection with either hyperuricemia or serum uric acid levels. Sex-stratified results are summarized in Table 4.

**Table 4:** Sex-stratified associations of *H. pylori* infection with hyperuricemia and serum uric acid levels.

Panel A. Hyperuricemia					
Sex	Model	N	PR	95% CI	*p* value
Male	Basic adjusted model	42,129	1.10	1.06–1.15	<0.001
Male	Extended clinical model	41,933	1.04	0.99–1.08	0.087
Female	Basic adjusted model	25,697	1.02	0.90–1.15	0.794
Female	Extended clinical model	25,589	0.96	0.85–1.08	0.509
P for *H. pylori* × sex interaction					0.110
**Panel B. Serum uric acid**					
**Sex**	**Model**	**N**	***β* (μmol/L)**	**95% CI**	***p* value**
Male	Basic adjusted model	42,129	5.23	3.38–7.08	<0.001
Male	Extended clinical model	41,933	2.60	0.86–4.35	0.004
Female	Basic adjusted model	25,697	2.88	0.84–4.93	0.006
Female	Extended clinical model	25,589	1.33	−0.59–3.26	0.174
P for *H. pylori* × sex interaction					0.765

Note: Table 4 presents two vertically arranged panels. Panel A reports PRs from modified Poisson regression with HC0 robust sandwich variance; Panel B reports *β* coefficients from OLS with HC3 heteroscedasticity-robust standard errors. Sex-stratified models were adjusted for age, BMI, SBP, FBG, TG, HDL-C, eGFR, ALT, GGT, and WBC. *p* values for interaction were estimated from full-population models including the *H. pylori* infection × sex term. CI, confidence interval; HUA, hyperuricemia; UA, uric acid; PR, prevalence ratio; BMI, body mass index; SBP, systolic blood pressure; FBG, fasting blood glucose; TG, triglycerides; HDL-C, high-density lipoprotein cholesterol; eGFR, estimated glomerular filtration rate; ALT, alanine aminotransferase; GGT, gamma-glutamyl transferase; WBC, white blood cell count.

### 3.5. Robustness and Supplementary Analyses

The robustness analyses were generally consistent with the extended clinical model. For hyperuricemia, the PR was 1.03 (95% CI: 0.99–1.07, *p* = 0.124) when serum creatinine replaced eGFR, 1.02 (95% CI: 0.98–1.07, *p* = 0.248) after excluding participants with eGFR < 60 mL/min/1.73 m², and 1.02 (95% CI: 0.98–1.06, *p* = 0.381) after excluding serum uric acid values outside the 1st–99th percentiles. Within the same hs-CRP complete-case subset (*n* = 25,828), the HUA estimate was virtually unchanged after addition of hs-CRP (PR approximately 0.990 before and after hs-CRP adjustment), indicating that the difference from the main-population estimate was primarily related to restriction to the hs-CRP-available subset rather than to hs-CRP adjustment itself. Robustness results are presented in Table 5 and S8 Table.

**Table 5:** Robustness analyses for the associations of *H. pylori* infection with hyperuricemia and serum uric acid levels.

Panel A. Hyperuricemia				
Analysis	N	PR	95% CI	*p* value
Creatinine replacing eGFR	67,522	1.03	0.99–1.07	0.124
Exclude eGFR < 60	67,144	1.02	0.98–1.07	0.248
Exclude UA outside 1st–99th percentile	66,171	1.02	0.98–1.06	0.381
Additional hs-CRP adjustment in hs-CRP complete-case subset	25,828	0.99	0.93–1.06	0.752
**Panel B. Continuous serum uric acid**				
**Analysis**	**N**	***β* (μmol/L)**	**95% CI**	***p* value**
Creatinine replacing eGFR	67,522	2.30	0.94–3.67	<0.001
Exclude eGFR < 60	67,144	2.09	0.74–3.43	0.002
Exclude UA outside 1st–99th percentile	66,171	1.73	0.47–2.99	0.007
Additional hs-CRP adjustment in hs-CRP complete-case subset	25,828	1.68	−0.53–3.88	0.136

Note: Table 5 presents two vertically arranged panels. Panel A reports PRs from modified Poisson regression with HC0 robust sandwich variance; Panel B reports *β* coefficients from OLS with HC3 heteroscedasticity-robust standard errors. The extended clinical model adjusted for age, sex, BMI, SBP, FBG, TG, HDL-C, eGFR, ALT, GGT, and WBC. In the creatinine sensitivity model, eGFR was replaced by serum creatinine. The hs-CRP sensitivity analysis was restricted to 25,828 participants with complete data for hs-CRP and all Model 4 covariates. CI, confidence interval; UA, uric acid; PR, prevalence ratio; BMI, body mass index; SBP, systolic blood pressure; FBG, fasting blood glucose; TG, triglycerides; HDL-C, high-density lipoprotein cholesterol; eGFR, estimated glomerular filtration rate; ALT, alanine aminotransferase; GGT, gamma-glutamyl transferase; WBC, white blood cell count; hs-CRP, high-sensitivity C-reactive protein.

For continuous serum uric acid, the robustness estimates were 2.30 μmol/L (95% CI: 0.94–3.67, *p* < 0.001) when creatinine replaced eGFR, 2.09 μmol/L (95% CI: 0.74–3.43, *p* = 0.002) after excluding eGFR < 60 mL/min/1.73 m^2^, and 1.73 μmol/L (95% CI: 0.47–2.99, *p* = 0.007) after excluding extreme serum uric acid values. In the hs-CRP complete-case subset, additional adjustment for hs-CRP yielded *β* = 1.68 μmol/L (95% CI: −0.53–3.88, *p* = 0.136). Robustness results are presented in Table 5 and S8 Table.

In an additional sensitivity analysis, all sequential models were refitted in the same Model 4 complete-case population (*n* = 67,522). For hyperuricemia, the PR decreased from 1.27 in Model 1 to 1.02 in Model 4, closely matching the estimates obtained from the model-specific complete-case analyses. For continuous serum uric acid, the corresponding *β* coefficient decreased from approximately 16 to 2.14 μmol/L. Thus, progressive attenuation persisted when the analysis population was held constant (S5 Table).

In addition, all covariates in the extended clinical model had VIF values below 2, indicating no substantial multicollinearity.

Functional-form sensitivity analyses yielded small HUA effect estimates across specifications. The linear Model 4 PR was 1.022 (95% CI: 0.983–1.064, *p* = 0.269), the 4-knot RCS sensitivity PR was 1.042 (95% CI: 1.003–1.082, *p* = 0.033), the 3-knot placement sensitivity PR was 1.044 (95% CI: 1.006–1.084, *p* = 0.024), and the joint log-transformed sensitivity PR was 1.018 (95% CI: 0.979–1.058, *p* = 0.371). These additional sensitivity analyses were conducted to examine model assumptions; no specification was selected on the basis of statistical significance.

## 4. Discussion

### 4.1. Principal Findings

In this large-scale health examination population (*N* = 67,826), we evaluated the cross-sectional association between *H. pylori* infection and abnormalities in uric acid metabolism. The crude PR for hyperuricemia was 1.27 and progressively attenuated to 1.02 in the fully adjusted modified Poisson model. The fully adjusted association between *H. pylori* positivity and hyperuricemia was small. For continuous serum uric acid, *H. pylori* positivity was associated with a statistically detectable but clinically modest *β* of 2.14 μmol/L. Sex interaction tests were not statistically significant. Functional-form sensitivity analyses showed that statistical significance for hyperuricemia varied across specifications, although effect estimates remained modest.

### 4.2. Attenuation after Adjustment for Metabolic, Renal, and Inflammatory Factors

In the present study, the apparent association between *H. pylori* infection and hyperuricemia was progressively attenuated after sequential adjustment. The unadjusted modified Poisson model yielded PR = 1.27, whereas the fully adjusted model yielded PR = 1.02 with a confidence interval including the null. This pattern indicates that the crude cross-sectional association does not necessarily represent an association independent of measured metabolic, renal, hepatic, and inflammatory factors. Rather, both conditions may coexist within a broader clinical context characterized by metabolic abnormalities, altered renal excretion, and low-grade systemic inflammation.

Previous studies have suggested potential links between *H. pylori* infection, insulin resistance, metabolic syndrome, and chronic low-grade inflammation, although the epidemiological evidence remains inconsistent [3]. Some observational studies and systematic reviews have reported that *H. pylori*-infected individuals are more likely to have metabolic disturbances and insulin resistance, whereas others have emphasized heterogeneity across populations and the influence of confounding factors [3,10,11]. In uric acid metabolism, insulin resistance and hyperinsulinemia may reduce renal uric acid excretion and promote urate reabsorption through effects on renal tubular urate transport [7,8]. These findings are compatible with overlapping metabolic and inflammatory characteristics, but the cross-sectional design cannot distinguish confounding from downstream pathways or establish a causal sequence.

The sequential attenuation observed across the model series is consistent with adjustment for measured clinical domains. After systolic blood pressure, fasting blood glucose, triglycerides, and HDL-C were included in Model 3, the HUA PR was substantially attenuated. With additional adjustment for eGFR, ALT, GGT, and WBC in Model 4, the PR was 1.02 and the confidence interval included the null. eGFR, calculated using the 2021 CKD-EPI equation, reflects renal capacity for uric acid clearance and is a relevant correlate of serum uric acid levels [6,13]. ALT and GGT are related to hepatic metabolic status and overall metabolic burden, whereas WBC reflects systemic inflammatory background.

Functional-form sensitivity analyses further qualified this interpretation. The HUA PR was 1.022 (95% CI: 0.983–1.064, *p* = 0.269) in the linear specification, 1.042 (95% CI: 1.003–1.082, *p* = 0.033) in the 4-knot spline sensitivity analysis, 1.044 (95% CI: 1.006–1.084, *p* = 0.024) in the 3-knot placement sensitivity analysis, and 1.018 (95% CI: 0.979–1.058, *p* = 0.371) in the joint log-transformation sensitivity analysis. Effect estimates remained small across specifications, although statistical significance was sensitive to the functional form used for continuous covariates. The corresponding continuous-UA estimates remained approximately 2–3 μmol/L across flexible and transformed specifications.

### 4.3. Sex-Stratified Findings and Clinical Relevance

Sex-stratified estimates were somewhat larger in men, but interaction tests were not statistically significant for either outcome. The fully adjusted difference in continuous serum uric acid among men was 2.60 μmol/L and should be interpreted as clinically modest.

These findings should therefore be interpreted cautiously and do not support a clearly sex-specific biological effect.

Men generally have higher baseline serum uric acid and hyperuricemia prevalence, potentially reflecting sex-related differences in renal urate handling and estrogen effects [14,15]. These background differences may contribute to the slightly stronger male estimate, but they do not establish sex-specific heterogeneity.

### 4.4. Strengths and limitations

This study has several strengths. First, it was based on a large health examination population, with 67,826 participants included in the main regression analyses, providing substantial statistical power. Second, a strict individual-level deduplication strategy was applied: for participants with multiple examinations during the study period, only the most recent valid examination record was retained, thereby reducing potential bias from repeated measurements. Third, we examined both hyperuricemia as a binary outcome and serum uric acid as a continuous outcome, so the findings did not rely solely on a single diagnostic threshold. Fourth, we used sequential adjustment models incorporating a broad range of objective health examination indicators, including demographic factors, BMI, blood pressure, glucose and lipid metabolism, renal function, liver enzymes, and WBC. Robustness analyses were also performed by replacing eGFR with serum creatinine, excluding participants with eGFR < 60 mL/min/1.73 m^2^, excluding extreme serum uric acid values, and additionally adjusting for hs-CRP in the hs-CRP-available subset. In addition, all covariates in the extended clinical model had low VIF values, indicating no substantial multicollinearity.

Several limitations should also be acknowledged. First, because of the cross-sectional design, *H. pylori* infection status, serum uric acid levels, and related metabolic indicators were measured at the same health examination. Therefore, temporal relationships could not be established, causal inference is not possible, and the findings should be interpreted as statistical associations rather than causal effects. Second, although multiple objective clinical indicators were adjusted for, residual confounding could not be fully excluded. The health examination database lacked information on several important lifestyle and medication-related factors, including high-purine diet, alcohol intake, particularly beer consumption, smoking, physical activity, and the use of medications that may substantially influence serum uric acid levels, such as diuretics, low-dose aspirin, and urate-lowering agents. Because these factors may be associated with both *H. pylori* infection and uric acid metabolism, residual confounding cannot be excluded and may have influenced the magnitude of the observed associations. Although our models included several objective clinical indicators related to metabolic and renal status, these variables could not fully replace direct information on lifestyle and medication use. Third, *H. pylori* infection status was determined using the ^13^C-urea breath test, which is commonly used to assess current infection. However, the retrospective database did not contain individual-level information on recent proton pump inhibitor, antibiotic, or bismuth use before ^13^C-UBT. Because these medications may reduce the sensitivity of *H. pylori* testing, some false-negative results and exposure misclassification cannot be excluded. Data on previous *H. pylori* infection and eradication history were also unavailable. Fourth, hs-CRP had substantial missingness in this cohort and was therefore not included in the primary models. WBC was retained as an inflammation-related marker in the main model, and hs-CRP was additionally adjusted for only in the available-subset sensitivity analysis. Incomplete control of inflammation and selection bias in the hs-CRP subset remain possible. Fifth, a substantial number of otherwise eligible participants lacked *H. pylori* test results, and participants entering the baseline analysis differed from excluded participants on several measured characteristics. Selection related to test availability therefore cannot be excluded. A further 4,566 participants were excluded because BMI was missing, and measured characteristics also differed between participants included in and excluded from the main regression population. The analytical database did not contain information on the operational reasons for test availability. Finally, the study population was derived from health examinations. Participants may have had greater health awareness or specific occupational backgrounds compared with the general community population, raising the possibility of a healthy worker effect. Therefore, caution is needed when generalizing these findings to the broader community or to specific clinical patient populations.

## 5. Conclusion

In this large health examination population, any remaining association between *H. pylori* positivity and hyperuricemia after comprehensive adjustment was small and sensitive to model specification. *H. pylori* positivity was also associated with a statistically detectable but clinically modest difference in continuous serum uric acid levels. Given the cross-sectional design, these findings should not be interpreted causally.

## Supporting information

S1 Table Complete regression results for the primary sequential models. (XLSX)

S2 Table Variable-level missingness in the study populations. (XLSX)

S3 Table Comparison of participants included in and excluded from the analysis populations. (XLSX)

S4 Table Logistic-regression sensitivity analysis for hyperuricemia. (XLSX)

S5 Table Same complete-case sequential models. (XLSX)

S6 Table Functional-form sensitivity analyses. (XLSX)

S7 Table Robust Wald tests for nonlinearity of continuous covariates. (XLSX)

S8 Table hs-CRP fixed-subset sensitivity analysis. (XLSX)

S9 Table Linear-model diagnostic and heteroscedasticity summary. (XLSX)

## Abbreviations

ALT: alanine aminotransferase
AST: aspartate aminotransferase
BMI: body mass index
BUN: blood urea nitrogen
CI: confidence interval
CKD-EPI: Chronic Kidney Disease Epidemiology Collaboration
DBP: diastolic blood pressure
eGFR: estimated glomerular filtration rate
FBG: fasting blood glucose
GGT: gamma-glutamyl transferase
HbA1c: glycated hemoglobin
Hcy: homocysteine
HDL-C: high-density lipoprotein cholesterol
Hp: *Helicobacter pylori*
hs-CRP: high-sensitivity C-reactive protein
HUA: hyperuricemia
IQR: interquartile range
LDL-C: low-density lipoprotein cholesterol
OR: odds ratio
PPI: proton pump inhibitor
Q1: first quartile
Q3: third quartile
SBP: systolic blood pressure
TC: total cholesterol
TG: triglycerides
UA: uric acid
VIF: variance inflation factor
WBC: white blood cell count
