# Supplementary material for: Helicobacter pylori infection, hyperuricemia, and serum uric acid levels: The role of metabolic, renal, and inflammatory factors in a large health examination population
Source: PLoS One. 2026 Sep 10;21(9):e0358039. doi: 10.1371/journal.pone.0358039 (PMC13561360; doi:10.1371/journal.pone.0358039)
Supplement: Supplementary file 1 [file pone.0358039.s011.docx]

# Response to the Editor and Reviewers

**Manuscript ID:** PONE-D-26-26394

**Manuscript title:** Helicobacter pylori infection, hyperuricemia, and serum uric acid levels: the role of metabolic, renal, and inflammatory factors in a large health examination population

Dear Editor and Reviewers,

Thank you for the careful and constructive evaluation of our manuscript. We carefully considered all comments and substantially revised the manuscript and accompanying materials. The revision includes participant-selection, missing-data, laboratory-method, and statistical analyses; modified Poisson prevalence-ratio modeling as the primary binary-outcome analysis; model-assumption and robustness assessments; a more cautious interpretation; final Supporting Information S1–S9; and public release of the complete author-generated analysis code. Locations are identified by section, table, figure, or Supporting Information item rather than provisional page and line numbers.

## Decision Letter / Editor-in-Chief

### DL-1

**Comment:**

Thank you for submitting your manuscript to PLOS ONE. After careful consideration, we feel that it has merit but does not fully meet PLOS ONE’s publication criteria as it currently stands. Therefore, we invite you to submit a revised version of the manuscript that addresses the points raised during the review process.

The manuscript has been evaluated by two reviewers, and their comments are available below. The reviewers note that a strength of the manuscript is the large sample size; however, they each raise concerns regarding the statistical analyses that must be fully addressed as a prerequisite to further consideration. In addition, the identity of the health examination database must be described in more detail.

Could you please carefully revise the manuscript to address all comments raised?

Please submit your revised manuscript by Oct 03 2026 11:59PM. If you will need more time than this to complete your revisions, please reply to this message or contact the journal office at plosone@plos.org. When you're ready to submit your revision, log on to <https://www.editorialmanager.com/pone/> and select the 'Submissions Needing Revision' folder to locate your manuscript file.

Please include the following items when submitting your revised manuscript:

A letter that responds to each point raised by the academic editor and reviewer(s). You should upload this letter as a separate file labeled 'Response to Reviewers'.

A marked-up copy of your manuscript that highlights changes made to the original version. You should upload this as a separate file labeled 'Revised Manuscript with Track Changes'.

An unmarked version of your revised paper without tracked changes. You should upload this as a separate file labeled 'Manuscript'.

**Response:**

We addressed the editor and reviewer comments through the revised manuscript, new aggregate analyses, expanded laboratory and database descriptions, revised Supporting Information, public code release, and this point-by-point response. The Track Changes and clean manuscript files have been generated and are included in the final submission package.

**Changes in the manuscript / submission materials:**

Whole submission package

## Journal Requirements

### JR-1

**Comment:**

Please ensure that your manuscript meets PLOS ONE's style requirements, including those for file naming. The PLOS ONE style templates can be found at <https://journals.plos.org/plosone/s/file?id=wjVg/PLOSOne_formatting_sample_main_body.pdf> and <https://journals.plos.org/plosone/s/file?id=ba62/PLOSOne_formatting_sample_title_authors_affiliations.pdf>.

**Response:**

We checked the manuscript against PLOS ONE style guidance, including the title page, section hierarchy, tables, figures, Supporting Information, and file labels. Final production file naming, continuous line numbering, and page numbering have been completed in the frozen submission package.

**Changes in the manuscript / submission materials:**

Title page; main text; tables; figures; supporting information

### JR-2

**Comment:**

Please note that PLOS One has specific guidelines on code sharing for submissions in which author-generated code underpins the findings in the manuscript. In these cases, we expect all author-generated code to be made available without restrictions upon publication of the work. Please review our guidelines at [https://journals.plos.org/plosone/s/materials-and-software-sharing#loc-sharing-code](https://journals.plos.org/plosone/s/materials-and-software-sharing#loc-sharing-code) and ensure that your code is shared in a way that follows best practice and facilitates reproducibility and reuse.

**Response:**

We complied with the code-sharing requirement by releasing the complete author-generated analysis code at <https://github.com/fangyong7462/PONE-D-26-26394-Hp-Uric-Acid>. Version v1.0.0 is archived at <https://doi.org/10.5281/zenodo.21868340>. Individual-level data are excluded because of privacy and institutional data-governance restrictions.

**Changes in the manuscript / submission materials:**

Data Availability / Code Availability; Supporting Information

### JR-3

**Comment:**

Thank you for stating in your Funding Statement:

“This work was supported in part by the 2022 National Key Research and Development Program of China, Key Special Project on “Active Health and Science and Technology Response to Population Aging” (No. 2022YFC3600903), entitled “Research on the Construction and Output Technology of Active Health Knowledge System.” The funder had no role in study design, data collection and analysis, decision to publish, or preparation of the manuscript. “

Please provide an amended statement that declares all the funding or sources of support (whether external or internal to your organization) received during this study, as detailed online in our guide for authors at <http://journals.plos.org/plosone/s/submit-now>. Please also include the statement “There was no additional external funding received for this study.” in your updated Funding Statement.

Please include your amended Funding Statement within your cover letter. We will change the online submission form on your behalf.

**Response:**

We prepared the amended Funding Statement and included it in the Cover Letter as requested. No additional internal or external support existed beyond the disclosed grant. The required sentence is retained verbatim: “There was no additional external funding received for this study.”

**Changes in the manuscript / submission materials:**

Cover Letter / online submission form

### JR-4

**Comment:**

In the online submission form, you indicated that your data is available only on request from a third party. Please note that your Data Availability Statement is currently missing [the name of the third party contact or institution / contact details for the third party, such as an email address or a link to where data requests can be made]. Please update your statement with the missing information.

**Response:**

The Data Availability Statement now identifies Shanghai Health and Medical Center and provides Hdlyyyb@126.com for institutional data-access requests, subject to institutional review and applicable data-use requirements. Code availability is stated separately.

**Changes in the manuscript / submission materials:**

Data Availability Statement

### JR-5

**Comment:**

Your ethics statement should only appear in the Methods section of your manuscript. If your ethics statement is written in any section besides the Methods, please delete it from any other section.

**Response:**

Ethics approval, the Declaration of Helsinki, and the informed-consent waiver are consolidated in Methods — Study Design and Data Source; the separate end-of-manuscript ethics section was removed.

**Changes in the manuscript / submission materials:**

Methods — Study Design and Data Source

### JR-6

**Comment:**

We note you have included a table to which you do not refer in the text of your manuscript. Please ensure that you refer to Table 2-5 in your text; if accepted, production will need this reference to link the reader to the Table.

**Response:**

Tables 2–5 are explicitly cited in the Results section in their corresponding analytical contexts.

**Changes in the manuscript / submission materials:**

Results — HUA, continuous UA, sex-stratified, and robustness analyses

### JR-7

**Comment:**

If the reviewer comments include a recommendation to cite specific previously published works, please review and evaluate these publications to determine whether they are relevant and should be cited. There is no requirement to cite these works unless the editor has indicated otherwise.

**Response:**

We reviewed the reviewer comments and identified no specific publication that the reviewers required us to cite. References were added or retained only when directly relevant to the issues raised.

**Changes in the manuscript / submission materials:**

Introduction / Discussion / References

## Additional Editor Comment

### AE-1

**Comment:**

As you revise your manuscript to address the reviewers' comments, please include in the Methods a description of the health examination database used in this research. If, for example, the database is composed of patient records from Shanghai Health and Medical Center (or another hospital or clinic), please state this in the Methods. If the study used third-party data (for example, the National Health and Nutrition Examination Survey (NHANES)) this also needs to be clearly indicated in the Methods.

**Response:**

Methods identifies the source as deidentified routine health-examination records maintained by Shanghai Health and Medical Center in Wuxi, Jiangsu Province, China, covering October 1, 2024 through October 31, 2025.

**Changes in the manuscript / submission materials:**

Methods — Study Design and Data Source

## Reviewer #1

### R1-1

**Comment:**

Please expand the discussion on residual confounding, especially because information on smoking, alcohol use, diet, physical activity, and medications was not available.

**Response:**

The Discussion acknowledges unavailable information on high-purine diet, alcohol and beer intake, smoking, physical activity, diuretics, low-dose aspirin, urate-lowering agents, and other medications. Residual confounding cannot be excluded.

**Changes in the manuscript / submission materials:**

Discussion — Strengths and Limitations

### R1-2

**Comment:**

The association found after adjustment is statistically significant but very small. Please emphasize that the clinical significance of this finding may be limited.

**Response:**

The manuscript emphasizes the fully adjusted difference of β=2.14 μmol/L (95% CI: 0.80–3.49, P=0.002) and describes it as statistically detectable but clinically modest.

**Changes in the manuscript / submission materials:**

Abstract; Results; Discussion; Conclusion

### R1-3

**Comment:**

Please provide a reference for the definition of hyperuricemia used in the study.

**Response:**

Methods cites Reference 12 for the conventional sex-specific epidemiological thresholds of >420 μmol/L in men and >360 μmol/L in women.

**Changes in the manuscript / submission materials:**

Methods — Outcome definition; References 12

### R1-4

**Comment:**

Please check the manuscript for small inconsistencies in percentages and table formatting.

**Response:**

Tables 1–5 and the corresponding Results text were audited for denominators, percentages, rounding, N values, estimates, confidence intervals, P values, units, footnotes, and variable-specific denominators.

**Changes in the manuscript / submission materials:**

Tables 1–5 and corresponding Results text

### R1-5

**Comment:**

Please ensure that the manuscript includes a clear Data Availability Statement in accordance with PLOS ONE requirements.

**Response:**

The manuscript now provides a clear Data Availability Statement with the institutional contact, access requirements, privacy restrictions, and separate public code information.

**Changes in the manuscript / submission materials:**

Data Availability Statement

## Reviewer #2 — Major Comments

### R2-1

**Comment:**

The authors frequently state that metabolic, renal, hepatic, and inflammatory factors “explain” the association. However, attenuation after adjustment in a cross-sectional analysis does not establish mediation or causality. Some adjusted variables may be confounders, whereas others could potentially lie on the pathway between infection and uric acid metabolism.

The authors should clarify the purpose of each model and replace causal wording such as “explained by” with more cautious language, for example:

“The association was substantially attenuated after adjustment for measured metabolic, renal, hepatic, and inflammation-related variables.”

The proposed “shared metabolic-inflammatory phenotype” should be presented as a possible interpretation rather than a demonstrated mechanism.

**Response:**

We agree that cross-sectional attenuation does not establish mediation or causality. Models 1–4 are described as domain-based sequential adjustment models, and the manuscript uses cautious association-based wording without a mediation claim.

**Changes in the manuscript / submission materials:**

Introduction; Methods — Covariates and Statistical Analysis; Discussion; Conclusion

## R2-2a

**Comment:**

The cohort decreased from 97,379 unique participants to 72,392 participants with valid H. pylori and uric acid data and then to 67,827 participants in the regression analyses.

The authors should provide a participant flow diagram and clarify:

**Response:**

Figure 1 was added and participant selection was clarified: 109,329 initial records; 106,345 study-period records; 106,344 valid study-period records; 97,378 unique participants; 72,392 baseline participants; and 67,826 main-regression participants. Figure 1 is maintained as a separate submission figure.

**Changes in the manuscript / submission materials:**

Methods — Participants; Results; Figure 1 / Supporting Information

## R2-2b

**Comment:**

The authors should provide a participant flow diagram and clarify:

whether the ^13C-urea breath test was routine or optional;

**Response:**

Methods states that the 13C-urea breath test was routinely performed as part of health-examination services rather than specifically for this study, and was not available for every examinee.

**Changes in the manuscript / submission materials:**

Methods — Participants / H. pylori assessment

## R2-2c

**Comment:**

The authors should provide a participant flow diagram and clarify:

why such a large number of participants lacked relevant measurements;

**Response:**

We quantified missing H. pylori and serum uric acid measurements and report variable-level missingness in S2 Table. The retrospective database did not record the operational reason why an H. pylori test result was unavailable for an individual examinee; this limitation and the possibility of selection related to test availability are now stated explicitly.

**Changes in the manuscript / submission materials:**

Methods — Participants / Missing data; Results

## R2-2d

**Comment:**

The authors should provide a participant flow diagram and clarify:

whether included and excluded participants differed;

**Response:**

Aggregate included-versus-excluded comparisons are reported in S3 Table, including the baseline-to-main-regression comparison of 67,826 included and 4,566 excluded participants.

**Changes in the manuscript / submission materials:**

Results; Supporting Information; Discussion — Limitations

## R2-2e

**Comment:**

The authors should provide a participant flow diagram and clarify:

and whether selection for H. pylori testing could have introduced bias.

**Response:**

The Discussion acknowledges possible selection bias related to H. pylori test availability and the absence of operational reasons for test availability in the database.

**Changes in the manuscript / submission materials:**

Discussion — Limitations

## R2-2f

**Comment:**

A table reporting missing data for each variable is required.

**Response:**

A variable-level missing-data table covering the deduplicated, baseline-eligible, and main-regression populations was added as S2 Table.

**Changes in the manuscript / submission materials:**

Methods — Missing data; Results; Supporting Information

## R2-2g

**Comment:**

The statement that missingness was below 1% for core covariates appears inconsistent with the exclusion of 4,565 participants after the baseline analysis.

**Response:**

The revised text reports BMI missingness as 4,566/72,392 (6.31%), with 67,826 main-regression participants, 67,540 Model 3 participants, and 67,522 Model 4 participants.

**Changes in the manuscript / submission materials:**

Methods — Missing data; Results; Discussion

## R2-2h

**Comment:**

Sequential models should also be repeated in the same complete-case population to ensure that attenuation is not partly caused by changing sample sizes.

**Response:**

Models 1–4 were refitted in the same Model 4 complete-case population (N=67,522) for both outcomes. Progressive attenuation persisted; results are provided in S5 Table.

**Changes in the manuscript / submission materials:**

Methods — Statistical Analysis; Results; Supporting Information

## R2-3a

**Comment:**

The test protocol is insufficiently described. The Methods should include:

manufacturer and analytical system;

**Response:**

Methods identifies the HY-IREXplus breath analyzer and Jiangxi Xinyu Lian’er Medical Technology Co., Ltd.

**Changes in the manuscript / submission materials:**

Methods — H. pylori assessment

## R2-3b

**Comment:**

The test protocol is insufficiently described. The Methods should include:

administered ^13C-urea dose;

**Response:**

Methods reports ingestion of 75 mg of 13C-urea granules with 80 mL of cool water.

**Changes in the manuscript / submission materials:**

Methods — H. pylori assessment

## R2-3c

**Comment:**

The test protocol is insufficiently described. The Methods should include:

timing of breath samples;

**Response:**

Methods reports baseline and second breath samples at 0 and 30 min, with approximately 4–5 seconds of exhalation into a sample bag at each collection.

**Changes in the manuscript / submission materials:**

Methods — H. pylori assessment

## R2-3d

**Comment:**

The test protocol is insufficiently described. The Methods should include:

positivity threshold;

**Response:**

Results were expressed as delta over baseline, calculated as the 30-min value minus baseline; DOB ≥4.0 was positive and DOB <4.0 negative.

**Changes in the manuscript / submission materials:**

Methods — H. pylori assessment

## R2-3e

**Comment:**

The test protocol is insufficiently described. The Methods should include:

fasting requirements;

**Response:**

Methods states that participants undergoing testing fasted for at least 8 h.

**Changes in the manuscript / submission materials:**

Methods — H. pylori assessment

## R2-3f

**Comment:**

The test protocol is insufficiently described. The Methods should include:

quality-control procedures;

**Response:**

We added the supported description that routine instrument self-checks were performed according to analyzer operating procedures, without inventing QC materials, frequencies, criteria, or external QA details.

**Changes in the manuscript / submission materials:**

Methods — H. pylori assessment

## R2-3g

**Comment:**

The test protocol is insufficiently described. The Methods should include:

and required withdrawal periods for proton pump inhibitors, antibiotics, and bismuth compounds.

**Response:**

Manufacturer documentation identified recent proton pump inhibitor, antibiotic, and bismuth use as potential sources of test interference. Individual medication history, withdrawal intervals, and adherence were unavailable; we therefore did not report unverified institutional withdrawal intervals.

**Changes in the manuscript / submission materials:**

Methods — H. pylori assessment; Discussion — Limitations

## R2-3h

**Comment:**

The potential influence of recent treatment on false-negative results should be discussed.

**Response:**

The Discussion states that recent medication use may reduce 13C-UBT sensitivity, so false-negative results and exposure misclassification cannot be excluded.

**Changes in the manuscript / submission materials:**

Discussion — Limitations

## R2-4a

**Comment:**

The manuscript should provide details regarding the measurement of serum uric acid, creatinine, glucose, lipids, liver enzymes, WBC, and hs-CRP, including analytical methods, equipment, reagents, fasting conditions, and quality control.

**Response:**

Methods reports fasting conditions, Roche cobas 8000 c702, matched Roche reagents, analyte-specific biochemical methods, Mindray BC-6800Plus/CAL 8000Plus hematology methods, and supported calibration/internal QC wording. The hs-CRP platform and Roche reagent manufacturer were confirmed; its exact analytical principle was not inferred.

**Changes in the manuscript / submission materials:**

Methods — Laboratory Measurements

## R2-4b

**Comment:**

The sex-specific hyperuricemia thresholds of >420 μmol/L in men and >360 μmol/L in women should be supported by an appropriate reference.

**Response:**

Reference 12 was added to support the sex-specific thresholds, and all subsequent in-text citations and reference numbers were reconciled accordingly.

**Changes in the manuscript / submission materials:**

Methods — Hyperuricemia definition; References

### R2-5

**Comment:**

The rationale for including some variables and excluding others is unclear. For example, the model includes BMI but not waist circumference, systolic but not diastolic blood pressure, fasting glucose but not HbA1c, and ALT and GGT but not AST.

The authors should explain whether covariates were selected based on prior evidence, a causal framework, data availability, or statistical criteria. They should also clarify whether the analysis plan was established before inspecting the results.

**Response:**

The original sequential adjustment framework was based on clinical domains, prior epidemiological evidence, data availability, parsimony, and avoidance of redundant correlated measures rather than outcome-driven stepwise selection. The study did not use a preregistered causal modeling framework. Additional modified Poisson, spline, transformation, heteroscedasticity-robust, and diagnostic analyses were introduced during revision to address the reviewers’ methodological concerns.

**Changes in the manuscript / submission materials:**

Methods — Covariates

## R2-6a

**Comment:**

Because this is a cross-sectional study, the manuscript should refer to prevalence or prevalence odds rather than “risk.”

**Response:**

Study-outcome risk terminology was replaced with prevalence, prevalence ratio, or cross-sectional association wording where appropriate.

**Changes in the manuscript / submission materials:**

Whole manuscript; Abstract, Results, tables, and footnotes screened

## R2-6b

**Comment:**

Since hyperuricemia prevalence is relatively high, adjusted prevalence ratios obtained using Poisson regression with robust variance may be easier to interpret than odds ratios.

**Response:**

The primary binary analysis is modified Poisson regression with log link and HC0 robust sandwich variance. Model 4 was PR=1.02 (95% CI: 0.98–1.06, P=0.269); logistic regression remains sensitivity analysis.

**Changes in the manuscript / submission materials:**

Methods — Statistical Analysis; Results; Supporting Information

## R2-6c

**Comment:**

The authors should also:

evaluate non-linear relationships of continuous covariates;

**Response:**

Functional-form sensitivity results were: linear PR=1.022 (P=0.269), 4-knot RCS PR=1.042 (P=0.033), 3-knot PR=1.044 (P=0.024), and joint log PR=1.018 (P=0.371). Estimates remained small, while significance was specification-sensitive.

**Changes in the manuscript / submission materials:**

Methods — Statistical Analysis; Results; Supporting Information

## R2-6d

**Comment:**

The authors should also:

assess residuals and heteroscedasticity in linear regression;

**Response:**

Continuous serum uric acid was analyzed using OLS with HC3 heteroscedasticity-robust standard errors; aggregate diagnostics are provided in S9 Table.

**Changes in the manuscript / submission materials:**

Methods — Statistical Analysis; Supporting Information

## R2-6e

**Comment:**

The authors should also:

assess residuals and heteroscedasticity in linear regression;

**Response:**

Residual and heteroscedasticity diagnostics were assessed without releasing individual-level residual or leverage records. Model 4 was β=2.14 μmol/L (95% CI: 0.80–3.49, P=0.002).

**Changes in the manuscript / submission materials:**

Methods — Statistical Analysis; Results / Supporting Information

## R2-6f

**Comment:**

The authors should also:

consider transformations for skewed variables;

**Response:**

The joint log-transformation sensitivity analysis used log-transformed FBG, TG, ALT, and GGT. Because hs-CRP was highly skewed and available only in a subset, raw and log-transformed hs-CRP were evaluated separately within the identical hs-CRP complete-case population.

**Changes in the manuscript / submission materials:**

Methods — Statistical Analysis; Results / Supporting Information

## R2-6g

**Comment:**

The authors should also:

and report the exact software packages and functions used.

**Response:**

Methods reports Python 3.13.9, pandas 2.3.3, NumPy 2.3.5, SciPy 1.16.3, statsmodels 0.14.5, and patsy 1.0.1; the public documentation provides the exact package/function inventory.

**Changes in the manuscript / submission materials:**

Methods — Statistical Analysis; Code README

## R2-6h

**Comment:**

The statement that regression estimates were generated using “matrix-based” routines is not sufficiently reproducible. The analyses should preferably be validated using established statistical software, and the code should be made available.

**Response:**

Aggregate results were reproduced with established statistical packages rather than custom matrix-based inference routines. Complete author-generated code, sanitized upstream logic, documentation, and aggregate expected results are publicly available at GitHub (<https://github.com/fangyong7462/PONE-D-26-26394-Hp-Uric-Acid>) and permanently archived in Zenodo (<https://doi.org/10.5281/zenodo.21868340>), version v1.0.0.

**Changes in the manuscript / submission materials:**

Methods — Statistical Analysis; Data/Code Availability; Supporting Information

## R2-7a

**Comment:**

The fully adjusted increase in serum uric acid was only 2.14 μmol/L, indicating very limited clinical relevance. This should remain central to the interpretation.

**Response:**

The 2.14 μmol/L fully adjusted continuous-UA difference remains central and is described as clinically modest.

**Changes in the manuscript / submission materials:**

Abstract; Results; Discussion; Conclusion

## R2-7b

**Comment:**

Moreover, after additional adjustment for hs-CRP, the association with continuous serum uric acid was no longer statistically significant. This result should be discussed more clearly. It may reflect additional inflammatory adjustment, reduced statistical power, selection bias in the hs-CRP subset, or a combination of these factors.

**Response:**

In the identical hs-CRP complete-case subset (N=25,828), the continuous serum uric acid estimate was β=1.65 μmol/L (95% CI: −0.56 to 3.85, P=0.144) without hs-CRP and β=1.68 μmol/L (95% CI: −0.53 to 3.88, P=0.136) with raw hs-CRP. With log-transformed hs-CRP, the estimate was β=1.85 μmol/L (95% CI: −0.35 to 4.05, P=0.099). Thus, restricting the analysis from the main population to the hs-CRP complete-case subset changed the estimate, whereas adding hs-CRP within the identical subset produced little additional change. The loss of statistical significance was primarily related to subset restriction, sample composition, and reduced precision rather than a large effect of hs-CRP adjustment. HUA showed the same pattern: PR=0.990 without hs-CRP, 0.990 with raw hs-CRP, and 0.991 with log-transformed hs-CRP. This was a sensitivity analysis, not a mediation analysis. Details are in S8 Table.

**Changes in the manuscript / submission materials:**

Methods; Results; Discussion — Sensitivity analysis

### R2-8

**Comment:**

The conclusion should be more cautious. The study supports substantial attenuation after adjustment but does not establish that H. pylori and hyperuricemia share a specific biological phenotype.

A more appropriate conclusion would be:

In this cross-sectional health examination population, the crude association between H. pylori positivity and hyperuricemia was substantially attenuated after adjustment for measured metabolic, renal, hepatic, and inflammation-related variables. A small adjusted difference in continuous serum uric acid remained, but its clinical relevance was limited. Longitudinal and eradication-based studies are required to clarify temporality and potential mechanisms.

**Response:**

The Abstract and Conclusion state that the remaining association was small and model-sensitive, the continuous-UA difference was clinically modest, and the cross-sectional design prevents causal interpretation. No shared biological phenotype is claimed.

**Changes in the manuscript / submission materials:**

Abstract — Conclusion; Conclusion

## R2-m1

**Comment:**

Replace “risk” with “prevalence,” “prevalence odds,” or “cross-sectional association.”

**Response:**

The manuscript uses prevalence, prevalence ratio, or cross-sectional association terminology for the study outcomes rather than risk language.

**Changes in the manuscript / submission materials:**

Whole manuscript

## R2-m2

**Comment:**

Use eGFR, not EGFR, consistently.

**Response:**

The human-readable manuscript, tables, footnotes, and abbreviation list use eGFR consistently.

**Changes in the manuscript / submission materials:**

Whole manuscript; tables; footnotes

## R2-m3

**Comment:**

Add a participant flow diagram.

**Response:**

Figure 1 was completed as a separate submission figure and its manuscript citation was verified.

**Changes in the manuscript / submission materials:**

Methods; Results; Figure / Supporting Information

## R2-m4

**Comment:**

Report standardized differences in Table 1, as very small differences may produce P<0.001 in such a large cohort.

**Response:**

Table 1 now reports absolute standardized mean differences with variable-specific denominators and a formula footnote.

**Changes in the manuscript / submission materials:**

Methods — Statistical Analysis; Table 1

## R2-m5

**Comment:**

Clarify the sample size and denominator for each analysis.

**Response:**

Analysis-specific N values and denominators are reported in Tables 1–5, Methods, Results, and frozen Supporting Information.

**Changes in the manuscript / submission materials:**

Methods; Results; all tables and figures

## R2-m6

**Comment:**

Report the number and percentage of participants with available hs-CRP data.

**Response:**

The manuscript reports hs-CRP availability as 25,928/67,826 (38.23%) and the fixed complete-case sensitivity population as N=25,828.

**Changes in the manuscript / submission materials:**

Methods; Results; missingness table; sensitivity table

## R2-m7

**Comment:**

Clarify whether participants using urate-lowering medication, diuretics, antibiotics, proton pump inhibitors, or bismuth were included.

**Response:**

Medication information was unavailable at the individual level; the listed medications were not used as exclusion criteria, and related limitations are stated.

**Changes in the manuscript / submission materials:**

Methods — Covariates; Discussion — Strengths and Limitations

## R2-m8

**Comment:**

Shorten the Discussion, particularly the repetitive interpretation of model attenuation.

**Response:**

The Discussion was condensed by removing repeated attenuation and sex-stratified interpretations while retaining clinical relevance and limitations.

**Changes in the manuscript / submission materials:**

Discussion

## R2-m9

**Comment:**

Expand the literature concerning previous studies of H. pylori, hyperuricemia, and gout.

**Response:**

We expanded the Introduction and Discussion to compare the directly relevant Chen et al. and Wang et al. studies by population, outcome, adjustment, and interpretation.

**Changes in the manuscript / submission materials:**

Introduction; Discussion; References 10–11

## R2-m10

**Comment:**

The manuscript would benefit from careful English-language editing.

**Response:**

We performed a careful English-language edit throughout the manuscript to improve grammar, clarity, concision, and terminology consistency while preserving scientific content and statistical results.

**Changes in the manuscript / submission materials:**

Whole manuscript

## Reviewer Form / Data Availability

### RF-1

**Comment:**

Have the authors made all data underlying the findings in their manuscript fully available?

The PLOS Data policy requires authors to make all data underlying the findings described in their manuscript fully available without restriction, with rare exception (please refer to the Data Availability Statement in the manuscript PDF file). The data should be provided as part of the manuscript or its supporting information, or deposited to a public repository. For example, in addition to summary statistics, the data points behind means, medians and variance measures should be available. If there are restrictions on publicly sharing data—e.g. participant privacy or use of data from a third party—those must be specified.

Reviewer #1: No

Reviewer #2: Yes

**Response:**

The Data Availability Statement explains the privacy and institutional restrictions, provides the institutional access route, and separately provides the public code repository and Zenodo DOI. Supporting Information contains aggregate results only.

**Changes in the manuscript / submission materials:**

Data Availability Statement; Code Availability; Supporting Information

Sincerely,

Yong Fang and Chunxing Liu

Corresponding Authors
