# Supplementary material for: Helicobacter pylori infection, hyperuricemia, and serum uric acid levels: The role of metabolic, renal, and inflammatory factors in a large health examination population
Source: PLoS One. 2026 Sep 10;21(9):e0358039. doi: 10.1371/journal.pone.0358039 (PMC13561360; doi:10.1371/journal.pone.0358039)
Supplement: Peer review history

# Peer review: Decision Letter 0

**DOI:** 10.1371/journal.pone.0358039.r001

**Authors:** Emily Chenette

5 Aug 2026

Dear Dr. Fang,

The manuscript has been evaluated by two reviewers, and their comments are available below. The reviewers note that a strength of the manuscript is the large sample size; however, they each raise concerns regarding the statistical analyses that must be fully addressed as a prerequisite to further consideration. In addition, the identity of the health examination database must be described in more detail.

Could you please carefully revise the manuscript to address all comments raised?

Please submit your revised manuscript by Oct 03 2026 11:59PM. If you will need more time than this to complete your revisions, please reply to this message or contact the journal office at plosone@plos.org. When you're ready to submit your revision, log on to https://www.editorialmanager.com/pone/ and select the 'Submissions Needing Revision' folder to locate your manuscript file.

- A letter that responds to each point raised by the academic editor and reviewer(s). You should upload this letter as a separate file labeled 'Response to Reviewers'.
- A marked-up copy of your manuscript that highlights changes made to the original version. You should upload this as a separate file labeled 'Revised Manuscript with Track Changes'.
- An unmarked version of your revised paper without tracked changes. You should upload this as a separate file labeled 'Manuscript'.

If you would like to make changes to your financial disclosure, please include your updated statement in your cover letter. Guidelines for resubmitting your figure files are available below the reviewer comments at the end of this letter.

If applicable, we recommend that you deposit your laboratory protocols in protocols.io to enhance the reproducibility of your results. Protocols.io assigns your protocol its own identifier (DOI) so that it can be cited independently in the future. For instructions see: https://journals.plos.org/plosone/s/submission-guidelines#loc-laboratory-protocols. Additionally, PLOS ONE offers an option for publishing peer-reviewed Lab Protocol articles, which describe protocols hosted on protocols.io. Read more information on sharing protocols at https://plos.org/protocols?utm_medium=editorial-email&utm_source=authorletters&utm_campaign=protocols.

As the corresponding author, your ORCID iD is verified in the submission system and will appear in the published article. PLOS supports the use of ORCID, and we encourage all coauthors to register for an ORCID iD and use it as well. Please encourage your coauthors to verify their ORCID iD within the submission system before final acceptance, as unverified ORCID iDs will not appear in the published article. *Only*  the individual author can complete the verification step; PLOS staff *cannot*  verify ORCID iDs on behalf of authors.

We look forward to receiving your revised manuscript.

Kind regards,

Emily Chenette

Editor in Chief

PLOS One

Journal Requirements:

When submitting your revision, we need you to address these additional requirements.

1. Please ensure that your manuscript meets PLOS ONE's style requirements, including those for file naming. The PLOS ONE style templates can be found at

https://journals.plos.org/plosone/s/file?id=wjVg/PLOSOne_formatting_sample_main_body.pdf and

https://journals.plos.org/plosone/s/file?id=ba62/PLOSOne_formatting_sample_title_authors_affiliations.pdf.

2. Please note that PLOS One has specific guidelines on code sharing for submissions in which author-generated code underpins the findings in the manuscript. In these cases, we expect all author-generated code to be made available without restrictions upon publication of the work. Please review our guidelines at https://journals.plos.org/plosone/s/materials-and-software-sharing#loc-sharing-code and ensure that your code is shared in a way that follows best practice and facilitates reproducibility and reuse.

3. Thank you for stating in your Funding Statement:

“This work was supported in part by the 2022 National Key Research and Development Program of China, Key Special Project on “Active Health and Science and Technology Response to Population Aging” (No. 2022YFC3600903), entitled “Research on the Construction and Output Technology of Active Health Knowledge System.” The funder had no role in study design, data collection and analysis, decision to publish, or preparation of the manuscript. “

Please provide an amended statement that declares *all* the funding or sources of support (whether external or internal to your organization) received during this study, as detailed online in our guide for authors at http://journals.plos.org/plosone/s/submit-now.  Please also include the statement “There was no additional external funding received for this study.” in your updated Funding Statement.

Please include your amended Funding Statement within your cover letter. We will change the online submission form on your behalf.

4. In the online submission form, you indicated that your data is available only on request from a third party. Please note that your Data Availability Statement is currently missing [the name of the third party contact or institution / contact details for the third party, such as an email address or a link to where data requests can be made]. Please update your statement with the missing information.

5. Your ethics statement should only appear in the Methods section of your manuscript. If your ethics statement is written in any section besides the Methods, please delete it from any other section.

6. We note you have included a table to which you do not refer in the text of your manuscript. Please ensure that you refer to Table 2-5 in your text; if accepted, production will need this reference to link the reader to the Table.

7. If the reviewer comments include a recommendation to cite specific previously published works, please review and evaluate these publications to determine whether they are relevant and should be cited. There is no requirement to cite these works unless the editor has indicated otherwise.

Additional Editor Comments (if provided):

1) As you revise your manuscript to address the reviewers' comments, please include in the Methods a description of the health examination database used in this research. If, for example, the database is composed of patient records from Shanghai Health and Medical Center (or another hospital or clinic), please state this in the Methods. If the study used third-party data (for example, the National Health and Nutrition Examination Survey (NHANES)) this also needs to be clearly indicated in the Methods.

[Note: HTML markup is below. Please do not edit.]

Reviewers' comments:

Reviewer's Responses to Questions

**Comments to the Author**

1. Is the manuscript technically sound, and do the data support the conclusions?

Reviewer #1: Yes

Reviewer #2: Partly

**********

2. Has the statistical analysis been performed appropriately and rigorously?

Reviewer #1: Yes

Reviewer #2: Yes

**********

3. Have the authors made all data underlying the findings in their manuscript fully available?

The PLOS Data policy

Reviewer #1: No

Reviewer #2: Yes

**********

4. Is the manuscript presented in an intelligible fashion and written in standard English?

Reviewer #1: Yes

Reviewer #2: Yes

**********

Reviewer #1: Thank you for the opportunity to review this manuscript. This is a well-conducted study with a large sample size and appropriate statistical analyses. The manuscript is generally well written, and the conclusions are supported by the results. I have a few suggestions that may improve the manuscript.

1- Please expand the discussion on residual confounding, especially because information on smoking, alcohol use, diet, physical activity, and medications was not available.

2- The association found after adjustment is statistically significant but very small. Please emphasize that the clinical significance of this finding may be limited.

3- Please provide a reference for the definition of hyperuricemia used in the study.

4- Please check the manuscript for small inconsistencies in percentages and table formatting.

5- Please ensure that the manuscript includes a clear Data Availability Statement in accordance with PLOS ONE requirements.

Overall, I believe the manuscript is scientifically sound and would be suitable for publication after these minor revisions.

Reviewer #2: Overall assessment

This manuscript evaluates the cross-sectional association between Helicobacter pylori infection, hyperuricemia, and serum uric acid levels in a large health examination population. The large sample size, use of the ^13C-urea breath test, sequential regression models, sex-stratified analyses, and sensitivity analyses are important strengths.

The main finding is that the crude association between H. pylori positivity and hyperuricemia becomes non-significant after adjustment for metabolic, renal, hepatic, and inflammation-related variables. A small association with continuous serum uric acid remains, but its magnitude is unlikely to be clinically relevant.

The study is potentially valuable, but several methodological and interpretative issues should be addressed. I recommend major revision.

Major comments

1. Interpretation of sequential adjustment

The authors frequently state that metabolic, renal, hepatic, and inflammatory factors “explain” the association. However, attenuation after adjustment in a cross-sectional analysis does not establish mediation or causality. Some adjusted variables may be confounders, whereas others could potentially lie on the pathway between infection and uric acid metabolism.

The authors should clarify the purpose of each model and replace causal wording such as “explained by” with more cautious language, for example:

“The association was substantially attenuated after adjustment for measured metabolic, renal, hepatic, and inflammation-related variables.”

The proposed “shared metabolic-inflammatory phenotype” should be presented as a possible interpretation rather than a demonstrated mechanism.

2. Participant selection and missing data

The cohort decreased from 97,379 unique participants to 72,392 participants with valid H. pylori and uric acid data and then to 67,827 participants in the regression analyses.

The authors should provide a participant flow diagram and clarify:

whether the ^13C-urea breath test was routine or optional;

why such a large number of participants lacked relevant measurements;

whether included and excluded participants differed;

and whether selection for H. pylori testing could have introduced bias.

The statement that missingness was below 1% for core covariates appears inconsistent with the exclusion of 4,565 participants after the baseline analysis. A table reporting missing data for each variable is required. Sequential models should also be repeated in the same complete-case population to ensure that attenuation is not partly caused by changing sample sizes.

3. Description of the ^13C-urea breath test

The test protocol is insufficiently described. The Methods should include:

manufacturer and analytical system;

administered ^13C-urea dose;

timing of breath samples;

positivity threshold;

fasting requirements;

quality-control procedures;

and required withdrawal periods for proton pump inhibitors, antibiotics, and bismuth compounds.

The potential influence of recent treatment on false-negative results should be discussed.

4. Laboratory methods

The manuscript should provide details regarding the measurement of serum uric acid, creatinine, glucose, lipids, liver enzymes, WBC, and hs-CRP, including analytical methods, equipment, reagents, fasting conditions, and quality control.

The sex-specific hyperuricemia thresholds of >420 μmol/L in men and >360 μmol/L in women should be supported by an appropriate reference.

5. Covariate selection

The rationale for including some variables and excluding others is unclear. For example, the model includes BMI but not waist circumference, systolic but not diastolic blood pressure, fasting glucose but not HbA1c, and ALT and GGT but not AST.

The authors should explain whether covariates were selected based on prior evidence, a causal framework, data availability, or statistical criteria. They should also clarify whether the analysis plan was established before inspecting the results.

6. Statistical analysis

Because this is a cross-sectional study, the manuscript should refer to prevalence or prevalence odds rather than “risk.”

Since hyperuricemia prevalence is relatively high, adjusted prevalence ratios obtained using Poisson regression with robust variance may be easier to interpret than odds ratios.

The authors should also:

evaluate non-linear relationships of continuous covariates;

assess residuals and heteroscedasticity in linear regression;

consider transformations for skewed variables;

and report the exact software packages and functions used.

The statement that regression estimates were generated using “matrix-based” routines is not sufficiently reproducible. The analyses should preferably be validated using established statistical software, and the code should be made available.

7. Clinical relevance and hs-CRP analysis

The fully adjusted increase in serum uric acid was only 2.14 μmol/L, indicating very limited clinical relevance. This should remain central to the interpretation.

Moreover, after additional adjustment for hs-CRP, the association with continuous serum uric acid was no longer statistically significant. This result should be discussed more clearly. It may reflect additional inflammatory adjustment, reduced statistical power, selection bias in the hs-CRP subset, or a combination of these factors.

8. Conclusions

The conclusion should be more cautious. The study supports substantial attenuation after adjustment but does not establish that H. pylori and hyperuricemia share a specific biological phenotype.

A more appropriate conclusion would be:

In this cross-sectional health examination population, the crude association between H. pylori positivity and hyperuricemia was substantially attenuated after adjustment for measured metabolic, renal, hepatic, and inflammation-related variables. A small adjusted difference in continuous serum uric acid remained, but its clinical relevance was limited. Longitudinal and eradication-based studies are required to clarify temporality and potential mechanisms.

Minor comments

Replace “risk” with “prevalence,” “prevalence odds,” or “cross-sectional association.”

Use eGFR, not EGFR, consistently.

Add a participant flow diagram.

Report standardized differences in Table 1, as very small differences may produce P<0.001 in such a large cohort.

Clarify the sample size and denominator for each analysis.

Report the number and percentage of participants with available hs-CRP data.

Clarify whether participants using urate-lowering medication, diuretics, antibiotics, proton pump inhibitors, or bismuth were included.

Shorten the Discussion, particularly the repetitive interpretation of model attenuation.

Expand the literature concerning previous studies of H. pylori, hyperuricemia, and gout.

The manuscript would benefit from careful English-language editing.

Recommendation

The study addresses an interesting question and benefits from a large sample size. However, participant selection, missing data, laboratory methods, statistical reproducibility, and interpretation of adjusted associations require clarification.

Recommendation: Major Revision

**********

what does this mean?). If published, this will include your full peer review and any attached files.

If you choose “no”, your identity will remain anonymous but your review may still be made public.

**Do you want your identity to be public for this peer review?**  For information about this choice, including consent withdrawal, please see our Privacy Policy

Reviewer #1: **Yes:** Zean Zefenkey

Reviewer #2: No

**********

[NOTE: If reviewer comments were submitted as an attachment file, they will be attached to this email and accessible via the submission site. Please log into your account, locate the manuscript record, and check for the action link "View Attachments". If this link does not appear, there are no attachment files.]

To ensure your figures meet our technical requirements, please review our figure guidelines: https://journals.plos.org/plosone/s/figures

You may also use PLOS’s free figure tool, NAAS, to help you prepare publication quality figures: https://journals.plos.org/plosone/s/figures#loc-tools-for-figure-preparation.

NAAS will assess whether your figures meet our technical requirements by comparing each figure against our figure specifications.

# Author comment: Author response to Decision Letter 1

**DOI:** 10.1371/journal.pone.0358039.r002

14 Aug 2026

A detailed point-by-point response to all editor and reviewer comments has been uploaded as a separate “Response to Reviewers” file. All comments have been addressed, and the corresponding revisions are shown in the marked-up manuscript.

Attachment Submitted filename: PONE-D-26-26394_RESPONSE_TO_REVIEWERS_FINAL_FORMATTED.docx

# Peer review: Decision Letter 1

**DOI:** 10.1371/journal.pone.0358039.r003

**Authors:** Ahmed Abdel Moneim

26 Aug 2026

Helicobacter pylori infection, hyperuricemia, and serum uric acid levels: the role of metabolic, renal, and inflammatory factors in a large health examination population

PONE-D-26-26394R1

Dear Dr. Fang,

We’re pleased to inform you that your manuscript has been judged scientifically suitable for publication and will be formally accepted for publication once it meets all outstanding technical requirements.

Within one week, you’ll receive an e-mail detailing the required amendments. When these have been addressed, you’ll receive a formal acceptance letter and your manuscript will be scheduled for publication.

An invoice will be generated when your article is formally accepted. Please note, if your institution has a publishing partnership with PLOS and your article meets the relevant criteria, all or part of your publication costs will be covered. Please make sure your user information is up-to-date by logging into Editorial Manager at Editorial Manager® and clicking the ‘Update My Information' link at the top of the page. For questions related to billing, please contact billing support.

If your institution or institutions have a press office, please notify them about your upcoming paper to help maximize its impact. If they’ll be preparing press materials, please inform our press team as soon as possible -- no later than 48 hours after receiving the formal acceptance. Your manuscript will remain under strict press embargo until 2 pm Eastern Time on the date of publication. For more information, please contact onepress@plos.org.

Kind regards,

Ahmed E. Abdel Moneim

Academic Editor

PLOS One

Additional Editor Comments (optional):

Reviewers' comments:

Reviewer's Responses to Questions

**Comments to the Author**

Reviewer #1: All comments have been addressed

Reviewer #2: All comments have been addressed

**********

2. Is the manuscript technically sound, and do the data support the conclusions?

Reviewer #1: Yes

Reviewer #2: Yes

**********

3. Has the statistical analysis been performed appropriately and rigorously?

Reviewer #1: Yes

Reviewer #2: Yes

**********

4. Have the authors made all data underlying the findings in their manuscript fully available?

The PLOS Data policy

Reviewer #1: Yes

Reviewer #2: Yes

**********

5. Is the manuscript presented in an intelligible fashion and written in standard English?

Reviewer #1: Yes

Reviewer #2: Yes

**********

Reviewer #1: (No Response)

Reviewer #2: The authors have carefully addressed the comments raised during the previous review, and the revised manuscript has been substantially improved, particularly with regard to the statistical analyses, methodological description, and interpretation of the findings. The major concerns have been adequately resolved.

I noted only a few very minor editorial inconsistencies in the revised/marked-up version, mainly related to sample-size notation, table numbering, and wording in table notes. These should be corrected during final proofreading.

Overall, I consider the manuscript suitable for publication in PLOS ONE after these minor editorial corrections.

**********

what does this mean?). If published, this will include your full peer review and any attached files.

If you choose “no”, your identity will remain anonymous but your review may still be made public.

**Do you want your identity to be public for this peer review?**  For information about this choice, including consent withdrawal, please see our Privacy Policy

Reviewer #1: No

Reviewer #2: No

**********

# Editor report: Acceptance letter

**DOI:** 10.1371/journal.pone.0358039.r004

**Authors:** Ahmed Abdel Moneim

PONE-D-26-26394R1

PLOS One

Dear Dr. Fang,

I'm pleased to inform you that your manuscript has been deemed suitable for publication in PLOS One. Congratulations! Your manuscript is now being handed over to our production team.

At this stage, our production department will prepare your paper for publication. This includes ensuring the following:

* All references, tables, and figures are properly cited

* All relevant supporting information is included in the manuscript submission,

* There are no issues that prevent the paper from being properly typeset

You will receive further instructions from the production team, including instructions on how to review your proof when it is ready. Please keep in mind that we are working through a large volume of accepted articles, so please give us a few days to review your paper and let you know the next and final steps.

Lastly, if your institution or institutions have a press office, please let them know about your upcoming paper now to help maximize its impact. If they'll be preparing press materials, please inform our press team within the next 48 hours. Your manuscript will remain under strict press embargo until 2 pm Eastern Time on the date of publication. For more information, please contact onepress@plos.org.

You will receive an invoice from PLOS for your publication fee after your manuscript has reached the completed accept phase. If you receive an email requesting payment before acceptance or for any other service, this may be a phishing scheme. Learn how to identify phishing emails and protect your accounts at https://explore.plos.org/phishing.

If we can help with anything else, please email us at customercare@plos.org.

Thank you for submitting your work to PLOS One and supporting open access.

Kind regards,

PLOS One Editorial Office Staff

on behalf of

Dr. Ahmed E. Abdel Moneim

Academic Editor

PLOS One
